# Supplementary material for: Exceptional subgenome stability and functional divergence in the allotetraploid Ethiopian cereal teff
Source: Nat Commun. 2020 Feb 14;11:884. doi: 10.1038/s41467-020-14724-z (PMC7021729; doi:10.1038/s41467-020-14724-z)
Supplement: Supplementary file 1 — Supplementary Information [file 41467_2020_14724_MOESM1_ESM.pdf]

Exceptional subgenome stability and functional divergence in the allotetraploid Ethiopian cereal teff

VanBuren *et al.*

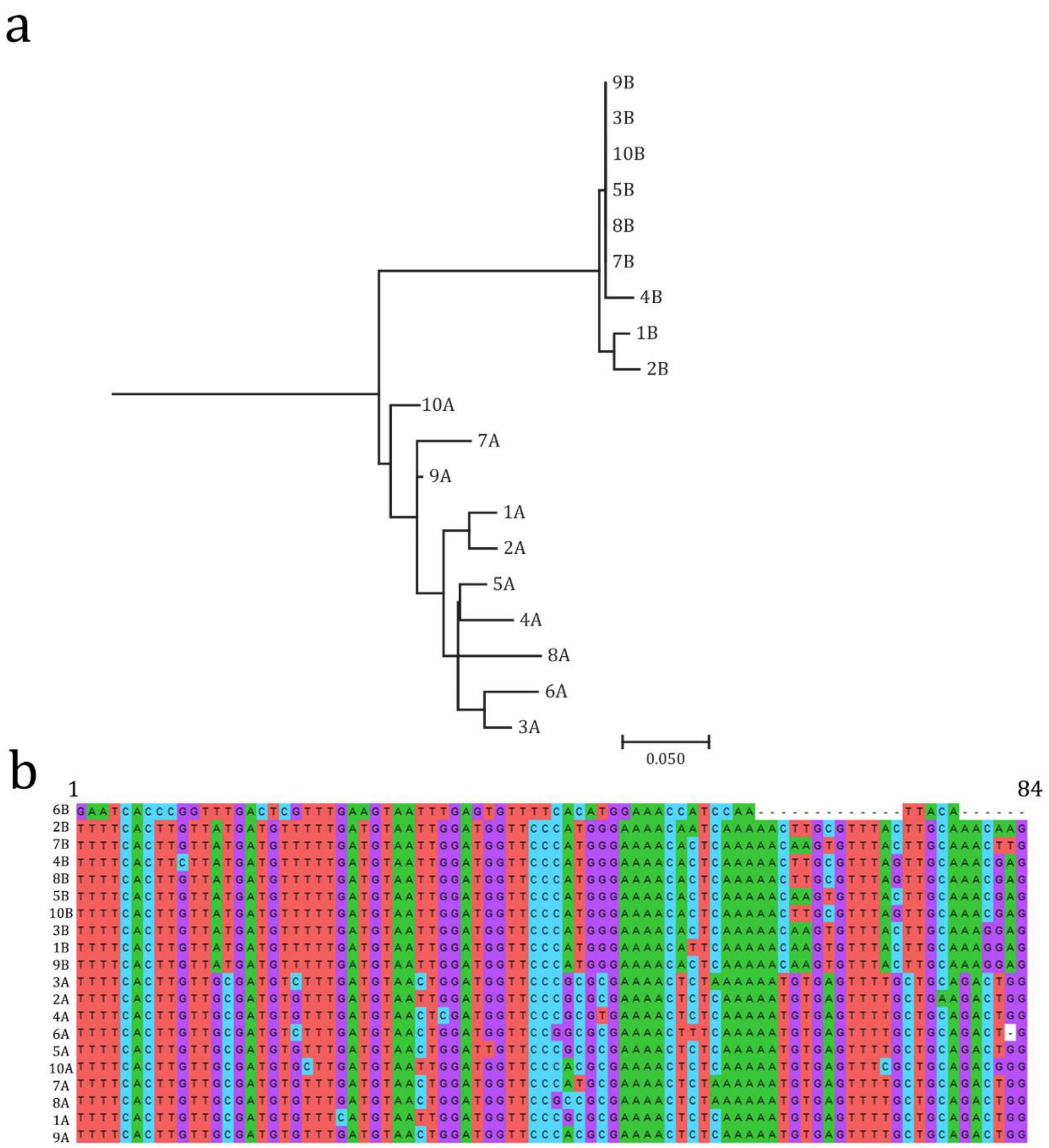

Supplementary Figure 1. Classification of putative centromeric repeat arrays in the A and B subgenomes. (a) Maximum likelihood phylogenetic tree of the consensus sequence for each of the 20 chromosomes. (b) Alignment of the SatTA and SatTB repeat arrays. A 84 bp subset of the 159 bp array with high homology is shown.

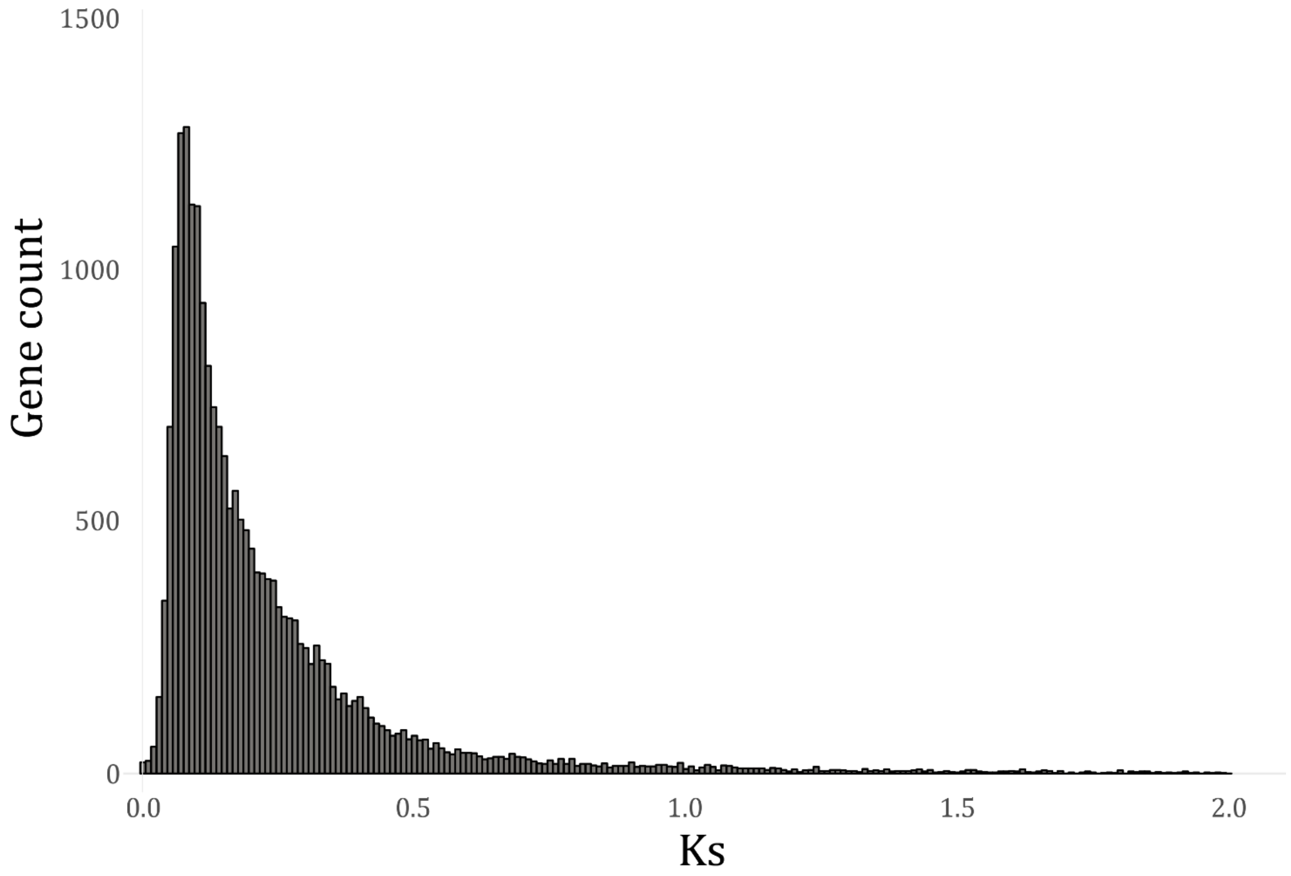

Supplementary Figure 2. Dating the divergence of the two subgenomes in tef. The distribution of Ks between homeologous gene pairs in the A and B subgenome is plotted. Source data are provided as a Source Data file.

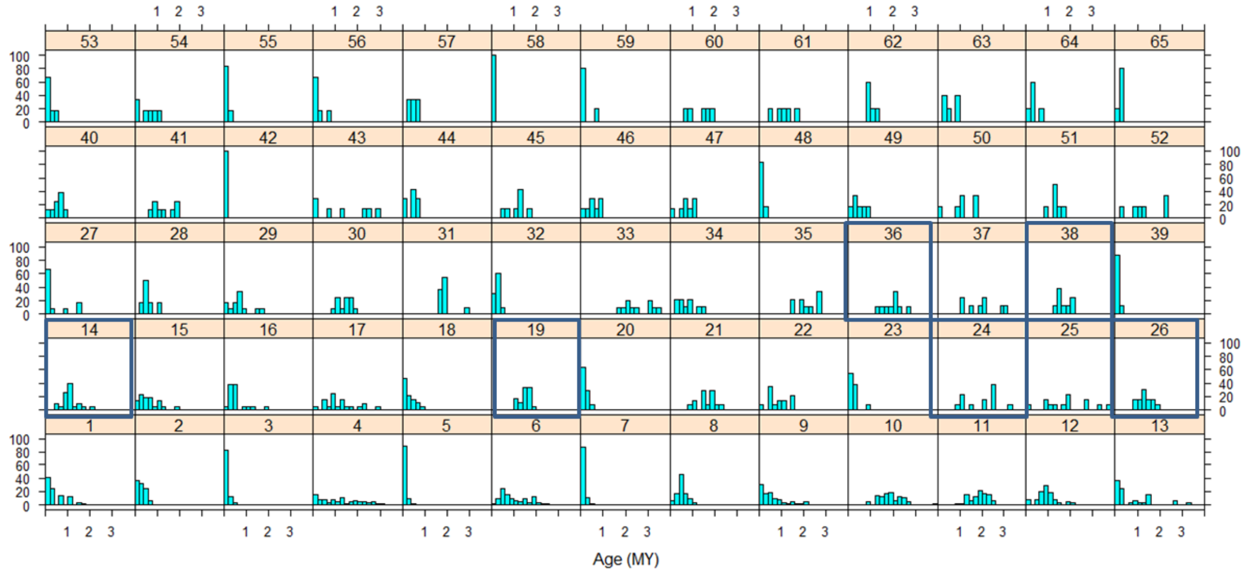

Supplementary Figure 3. Histogram of insertion times of 64 LTR families that having >= 5 intact LTR elements. In each panel, the Y-axis shows percentage and X-axis shows insertion time. The 6 subgenomic specific families are marked by blue blocks. Bin width = 0.2 MY. Source data are provided as a Source Data file

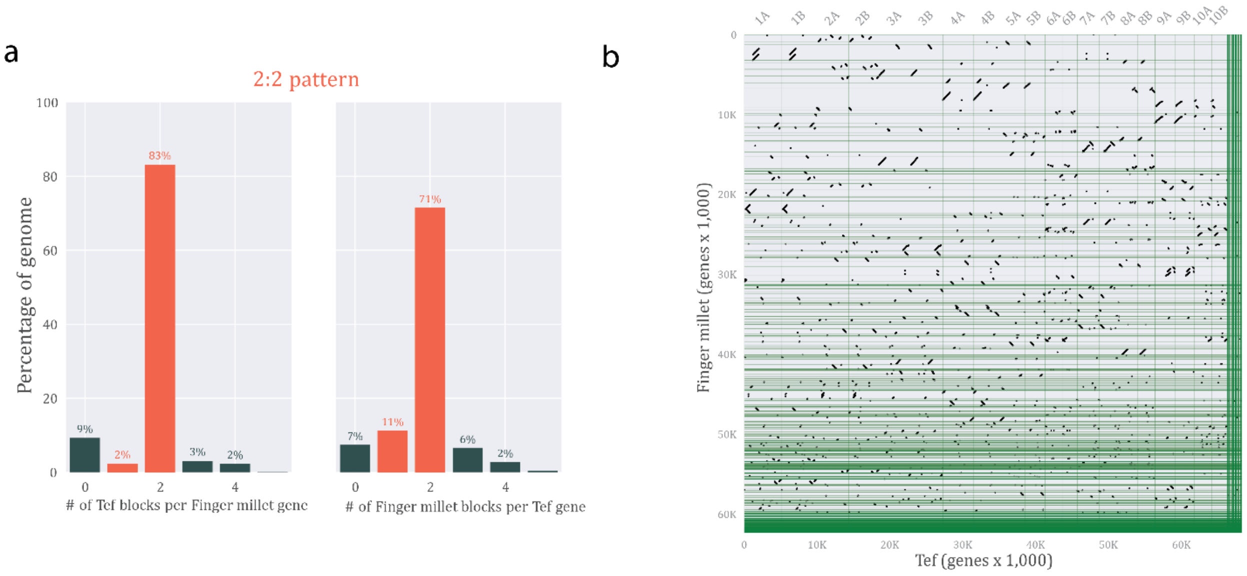

Supplementary Figure 4. Comparative genomics between the allotetraploid tef and Finger millet genomes. (a) Syntenic depth of tef blocks (left) and finger millet blocks (right) per finger millet and tef gene respectively. Syntenic blocks show a clear 2:2 pattern. (b) Macrosyntenic dotplot of the finger millet and tef genomes where each grey dot represents a syntenic gene pair. Source data underlying Supplementary Figure 4a are provided as a Source Data file.

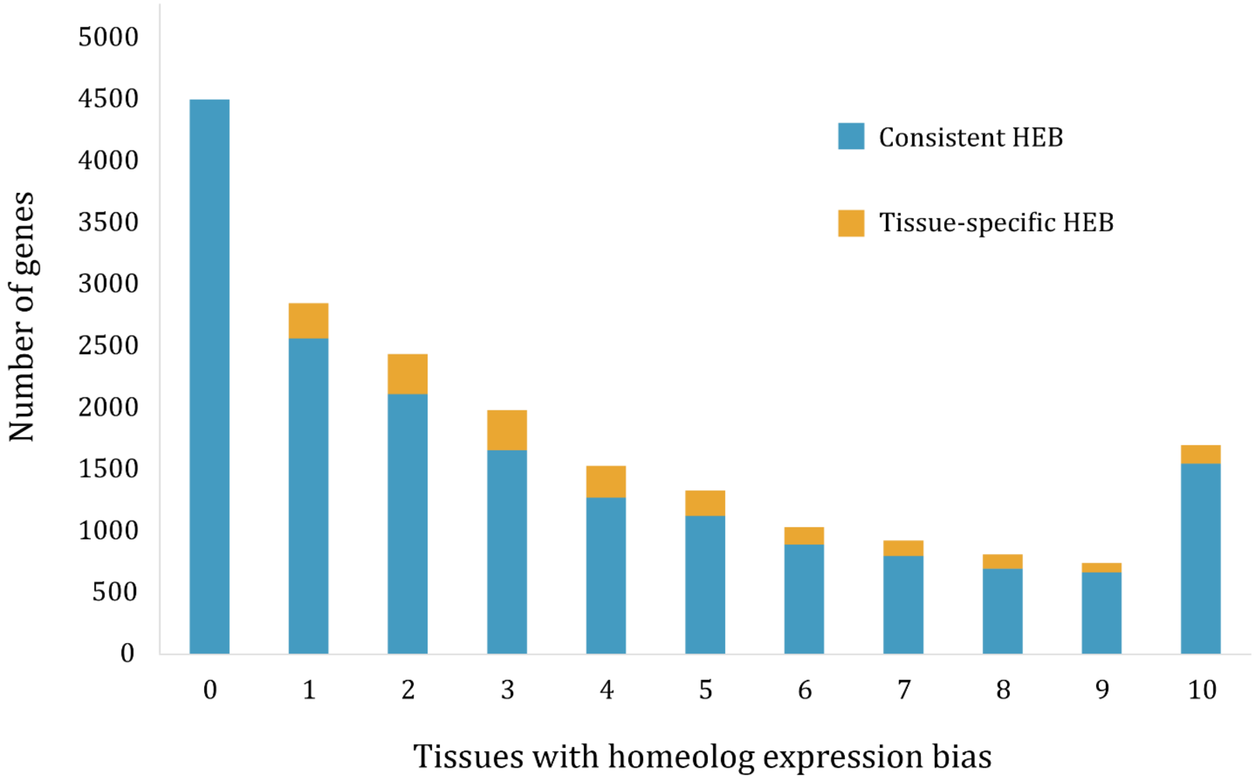

Supplementary Figure 5. Conservation of homeolog expression bias across tissues. A histogram showing the distribution of tissues showing homeolog expression bias (HEB) is plotted. Gene pairs showing consistent HEB are plotted in blue and genes with bias in both the A and B genomes in different tissues (tissue specific HEB) are shown in yellow. Source data are provided as a Source Data file.

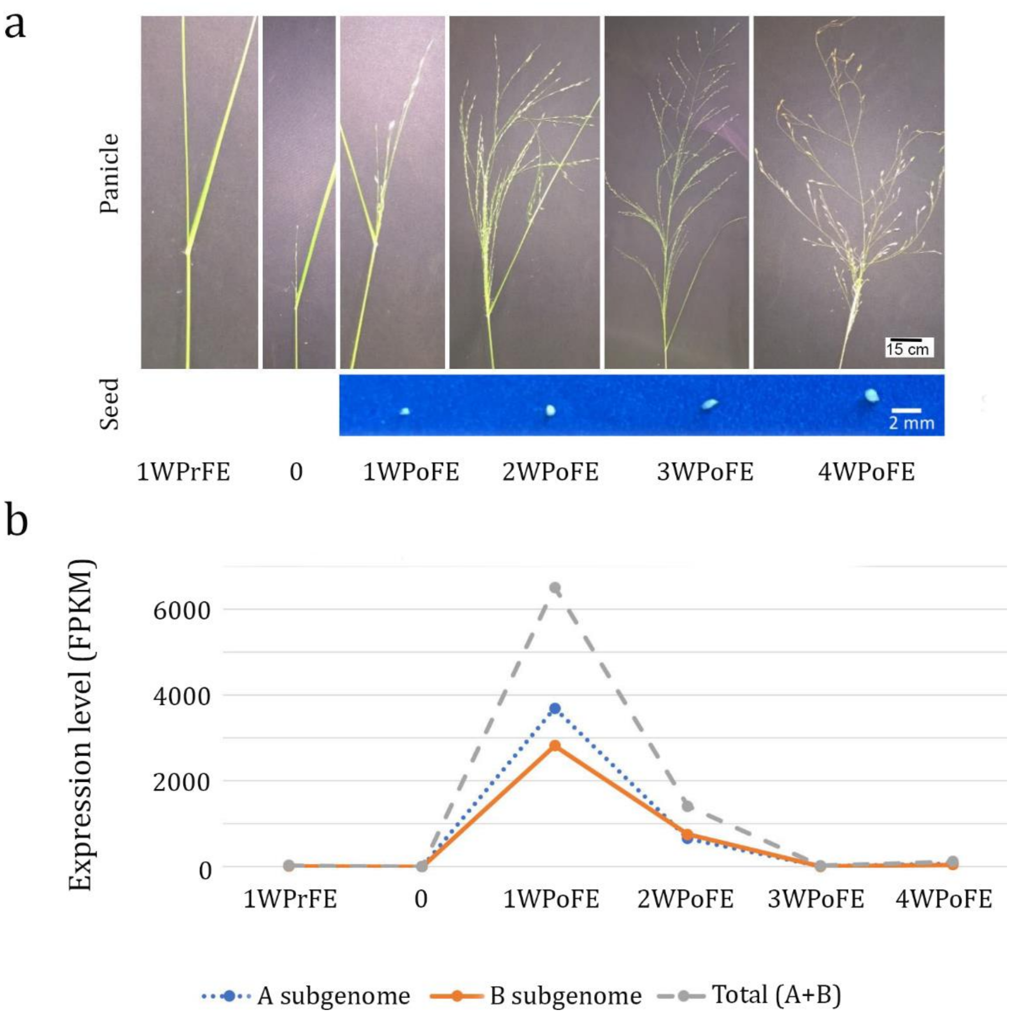

Supplementary Figure 6. Time-course of seed storage protein expression during seed development. (a) Images of panicle and seed development as weeks pre flower emergence (1WPrFE), at the time of flower emergence (0) and weeks post flower emergence (WPoFE). The curves in (b) shows the trend of total expression level of seed storage genes measured by RNA-Seq in FPKM. Three biological replicates were pooled for each seed developmental timepoint and sequenced as a single data point.

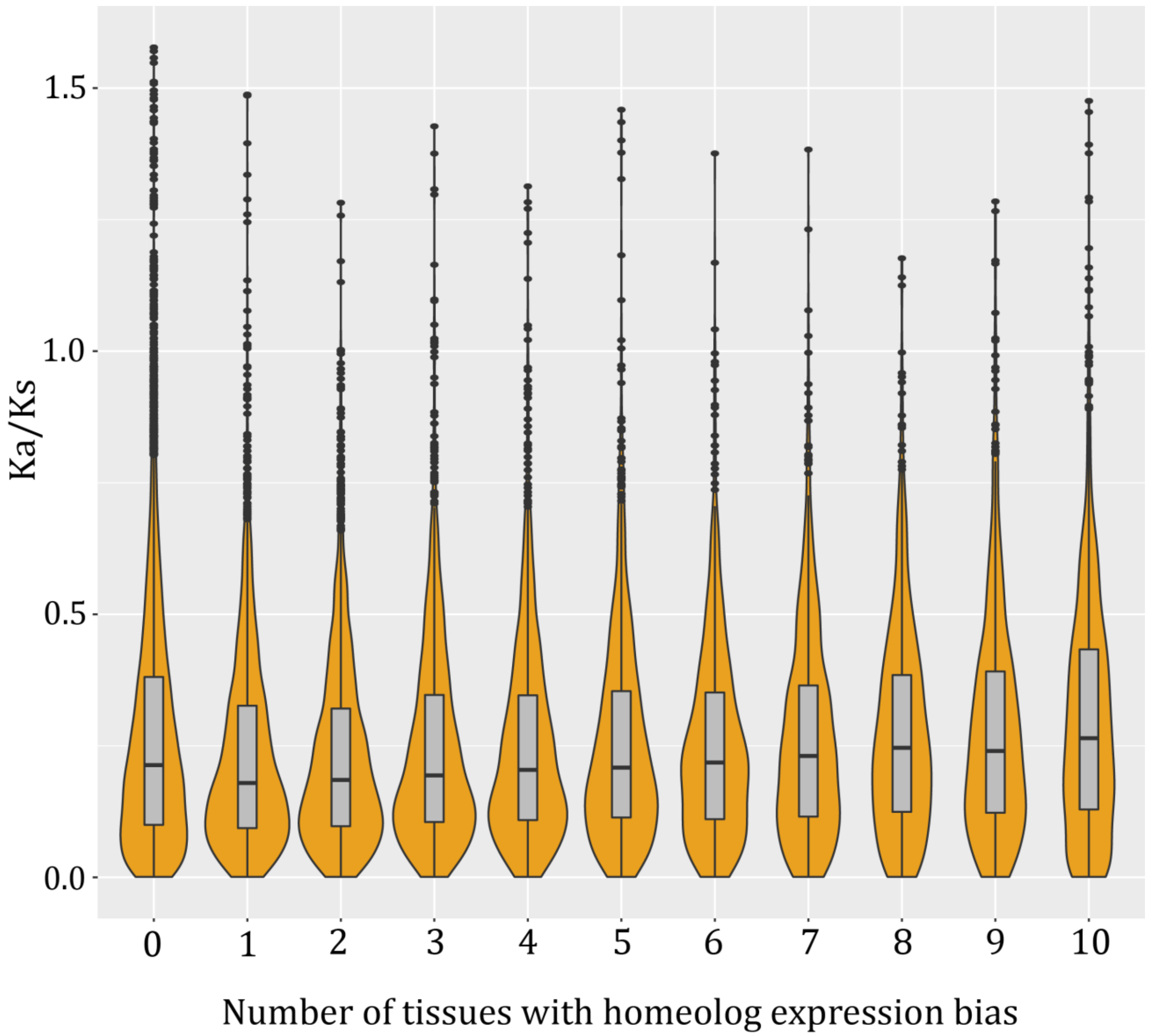

Supplementary Figure 7. Patterns of selective constraint and homeolog expression bias. Nested violin and box plots of Ka/Ks are shown for gene pairs ranging from 0 to 10 tissues with homeolog expression bias (HEB). Box boundaries indicate the 25th and 75th percentiles and whiskers extend to 1.5 times the interquartile range. Source data are provided as a Source Data file.

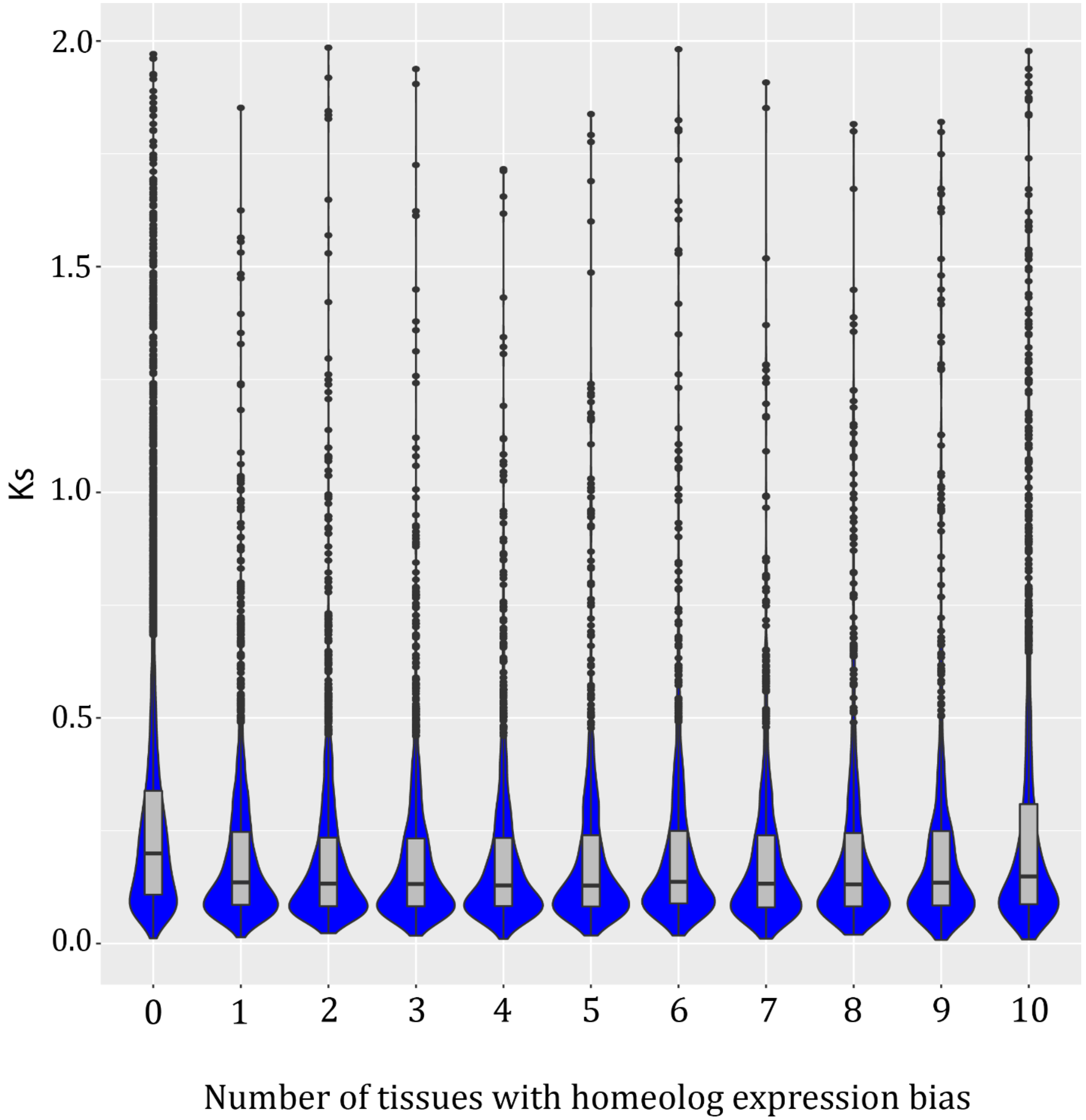

Supplementary Figure 8. Divergence of gene pairs and homeologous expression bias. Nested violin and box plots of divergence (Ks) are shown for gene pairs ranging from 0 to 10 tissues with HEB. Box boundaries indicate the 25th and 75th percentiles and whiskers extend to 1.5 times the interquartile range. Source data are provided as a Source Data file.

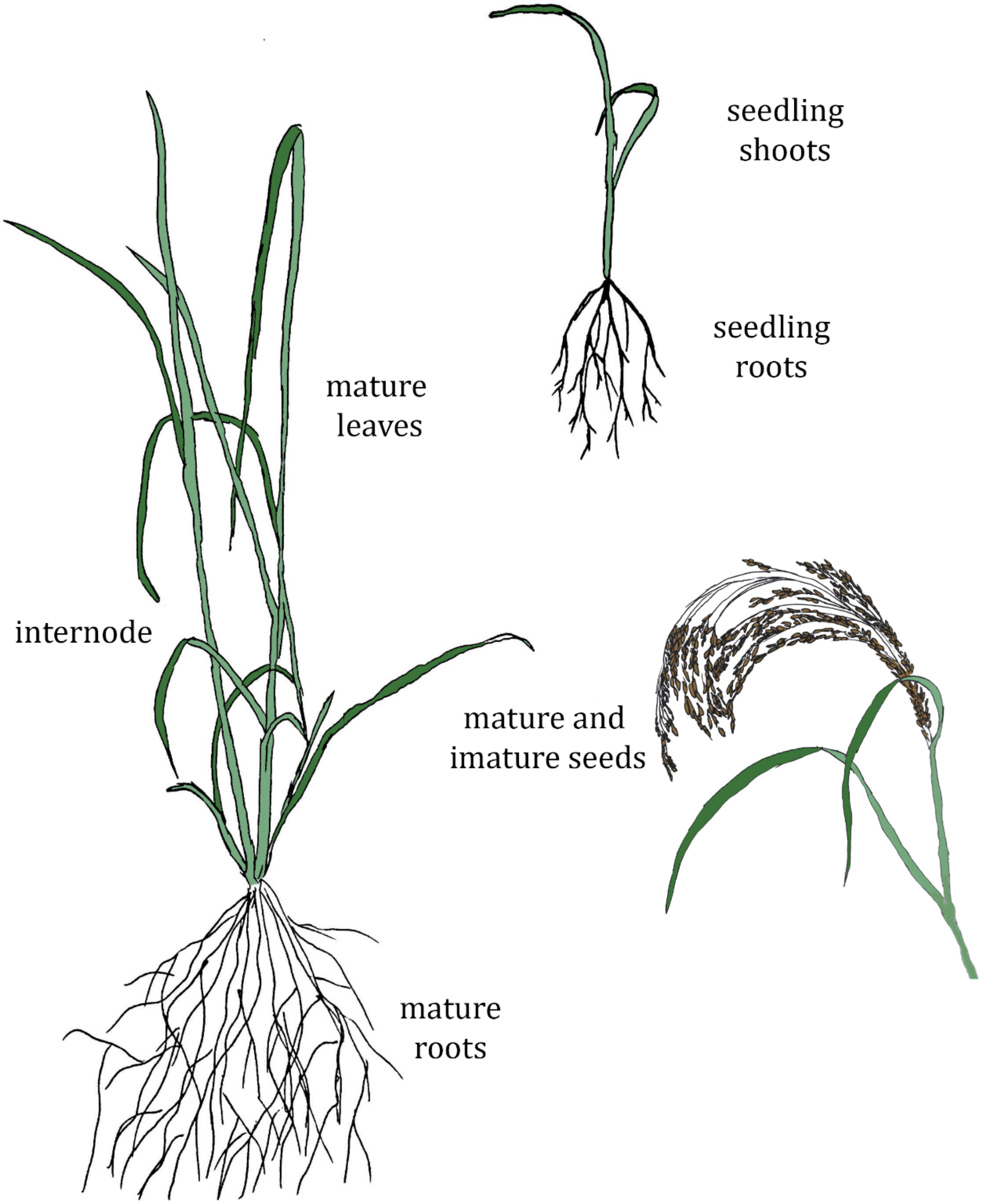

Supplementary Figure 9. Diagram of sampled tissues for the teff expression atlas. Tissues from shoots and roots of young seedlings, mature leaf, internode, and root, and immature and mature seeds were collected. Two timepoints of extreme drought were also collected (not pictured).

Supplementary Table 1. Summary of Fosmid alignment statistics to the tef genome.

Fosmid	Tef Chromosome	Pos. start	Pos. end	Length (bp)	Mismatches (indels/SNPs)	% identity
1	Chromosome_2A	24293453	24310772	17319	35	99.80
2	Chromosome_3B	480482	496500	16018	1	99.99
3	Chromosome_1A	607984	629933	21949	1	99.99
4	Chromosome_2B	1768602	1784819	16217	55	99.66
5	Chromosome_6B	9971798	9982526	10728	71	99.34
6	Chromosome_7A	18560345	18578197	17852	47	99.74
7	Chromosome_1A	607984	645075	37091	0	100
8	Chromosome_2A	13187152	13197778	10626	3	99.97
9	Chromosome_1A	629935	645075	15140	0	100
10	Chromosome_8A	7379205	7393614	14409	11	99.92
11	Chromosome_3B	467929	480309	12380	0	100
12	Chromosome_6B	19022180	19030707	8527	0	100
13	Chromosome_3A	15765021	15822822	57801	77	99.87
14	Chromosome_8B	2395467	2406855	11388	6	99.95
15	Chromosome_2A	25337979	25354980	17001	0	100
16	Chromosome_4B	4698711	4709012	10301	1	99.99
17	Chromosome_7B	1858973	1878530	19557	58	99.70
18	Chromosome_3A	23352162	23364268	12106	1	99.99
19	Chromosome_5A	18308369	18320474	12105	30	99.75
20	Chromosome_1A	35965810	35978550	12740	14	99.89
			Total	351255	411	99.9

Supplementary Table 2. Summary of centromeric repeat array composition in the tef genome

Chromosome	CenT[*] Array start	CenT Array End	CenT Array Length (bp)	Number of CenT repeats
1A	21,557,537	21,623,717	66,180	399
1B	19,501,629	19,520,995	19,366	114
2A	14,451,025	14,548,454	97,429	569
2B	12,104,147	12,108,587	4,440	26
3A	14,292,984	14,296,855	3,871	24
3B	14,092,648	14,176,019	83,371	121
4A	17,363,887	17,401,206	37,319	99
4B	15,985,907	16,152,093	166,186	824
5A	15,924,190	15,941,051	16,861	103
5B	13,811,928	13,872,280	60,352	115
6A	12,945,587	13,004,019	58,432	348
6B	8,377,013	8,380,775	3,762	22
7A	21,934,795	22,036,202	101,407	449
7B	21,827,654	21,831,745	4,091	24
8A	14,254,377	14,301,695	47,318	398
8B	12,502,475	12,627,067	124,592	117
9A	9,554,342	9,614,054	59,712	189
9B	7,617,457	7,629,471	12,014	71
10A	13,918,716	14,165,131	246,415	347
10B	10,984,530	11,310,738	326,208	231

[*] CenT: centromeric repeat array

Supplementary Table 3. Subgenome specificity of LTR-Retrotransposon.

Famly ID	Subgenome	No. of intact LTR-RT	Mean age	SD	Q25	Median age (MYA)	Q75
14	A	23	1.1	0.3	1.0	1.1	1.2
19	A	18	1.5	0.2	1.3	1.5	1.7
24	A	13	2.0	0.7	1.2	2.2	2.4
26	B	13	1.3	0.3	1.1	1.4	1.5
36	A	9	2.0	0.4	1.7	2.1	2.1
38	A	8	1.7++++++++	0.3	1.5	1.6	2.0

Supplementary Table 4. Summary of LTR-Retrotransposon subgenome specificity.

Category	No. of families
Single burst < 1 MYA	29
Not showing burst, active period upper bound < 1 MY	6
Not showing burst, active period upper bound > 1 MY	4
Two bursts, one < 1MY, the other > 1 MY	2
Not showing burst, active period upper bound >= 2 MY	15
Single burst	3
