# Supplementary material for: Exceptional subgenome stability and functional divergence in the allotetraploid Ethiopian cereal teff
Source: Nat Commun. 2020 Feb 14;11:884. doi: 10.1038/s41467-020-14724-z (PMC7021729; doi:10.1038/s41467-020-14724-z)
Supplement: Supplementary file 2 — Peer Review [file 41467_2020_14724_MOESM2_ESM.pdf]

Reviewers' comments:

Reviewer #1 (Remarks to the Author):

In this paper VanBuren et al present and describe a new genome assembly for the polyploid species Eragrostis teff. This chromosome scale genome assembly is based on PacBio reads along with HiC data and represents a great improvement in comparison to the previously available illumina based draft assembly (Cannarozzi et al, 2014). Indeed the statistics of completeness and contiguity for this assembly are impressive. Furthermore, using genome counterpart specific repetitive sequences the authors were able to associate each chromosome to the original donor genome (A or B). Analyzing the insertion time of TEs and their genome specificity the Authors were also able to estimate that the polyploidization event took place ~1.1 MYA. Analyzing the Ks between homeologus genes in E. tef the authors estimated that the two donor subgenomes diverged ~5.0 MYA. No major genome rearrangements were detected indicating an exceptional genome stability. Authors then provided an extensive survey of tef transcriptome showing divergent expression patterns among homeologous gene pairs.
Definitely this is a great resource for those interested in tef and more generally in orphan crops. Also it is a nice piece of work providing interesting insights in polyploid genome structure and functioning. Nevertheless there are few issues and a certain lack of detail that should be addressed by authors.
-First I would like to have the general statistics regarding repeats and TEs content and distribution moved to the main text (at present these data are provided in Supplemental Table 3). Indeed, as it clearly appears from the methods section, TEs annotation was carried out thoroughly but no general data are presented. Just a simple table clearly stating the amount of LTR-RT (possibly detailed for Ty1-copia and Ty3-gypsy), LINEs, SINEs etc… would be useful. I know that most part of this information is in main text and in supplemental data but I think that having it also in a single table in main manuscript would be beneficial for the readers.
-Then always related to TEs and in particular to those families seemingly specific for genome donors, all the period going from "We classified..." to "Supplemental table 5" (lines 177-192) is confusing and should be better described since it has a pivotal role in this manuscript. More in detail, it seems to me that authors dated the polyploidization event at 1.1 MYA (or before) basically relying on two facts: 1) all the species specific families (six) should have amplified before polyploidization and 2) the estimated insertion time is > 1.1 MYA for all the elements belonging to these families. I do not know if I got the reasoning correctly here. If not please apologize and explain. Anyway if this is correct then there is another simple test that authors should carry out to further support their conclusion. Consider out of the "24 families with median insertion times between 1.1 and 2.4 MYA" those that "do not exhibit subgenomic specificity". In this case, if you build a phylogenetic tree separately for PostPplyploidization (IT < 1.1 MYA) and PrePolyploidization (IT > 1.1 MYA) inserted elements then, for the first group elements should mix up whereas for the second subset they should separate in two distinct clades. Some families that could be considered for this exercise are 4, 6, 15, 16, 17, 25, 41, 43, 45, 52, 60 and 61.
Authors then stated that "The insertion times for these genome-specific LTR-RTs were all greater than 1.1 mya, indicating the two subgenomes were evolving independently during this period" but this doesn't seem to be the case of Family 14 that shows evidence of recent activity (supplemental figure 4).
Also authors concluded that because "Five of the six subgenome-specific LTR-RT families were found only in the A subgenome" then "LTR-RTs accumulate more rapidly in the A subgenome or are purged more effectively in the B subgenome." I am not completely sure about this. These are just subfamilies and authors are using their distribution pattern to infer the activity for the whole set of familyes: 65.
Other more specific points to fix or clarify are:

Line 84: is there a particular reason for focusing on "Dabbi" cv.? If there is, briefly explain it.
Lines 91-92: Authors should better specify the evolutionary relationship between E. tef and Oropetium thomaemum. How close is "closely related"?
Lines 99-102: from " Illumina reads from..." to "...between homoeologous regions". All this part

should be moved to Materials and Methods
Lines 115-117: It doesn't seem that the incongruences in figure Figure 2, chr 8A are negligible. Quite likely they could be explained somehow... but "negligible" seems to me an understatement.
Lines 142-144: These satellite sequences could well be centromeric satellite sequences. However it is important to be aware that the definition of centromeric sequence could not be based only on length and tandem arrangement. Here no prove is provided regarding the actual localization (FISH) and function (ad hoc biochemical essay) of these sequences although the analysis of co occurrence with centromeric specific Ty3-gypsy elements is a reasonable proxy. For these reasons I think that these sequences are for sure satellite sequences but they are only putatively located in centromeres. Because of this I would suggest to change CentT in SatT.
Lines 148-149: "This element was previously identified in the draft teff genome". Actually it was not identified in the draft teff sequence but in a set of random sheared MiSeq reads collected from a third Teff variety: Enantite
Line 158: "The Teff subgenomes have 93.9% sequence homology". It is not "93.9% homology" but "93.9% similarity". Homology by definition means: "sharing a common ancestor" and so it can not be quantified as a percentage. It is there or it is not.
Lines 163-164: mention the mutation rate here (not just the reference)
Line 166: Table 1. In this table it would be nice to have listed also the TEs content for each chromosome.
Lines 177-178: "We classified LTRs into families" please explicit that these families are 65.
Line 202: "Teff and Oropetium show a clear 1:2 synteny pattern" Shouldn't it be the opposite, i.e. a 2:1 pattern?
Lines 217-221. Supplemental table 6. Why 0.38 was chose as cutoff? Also list in supplemental table 6 the actual Ka/Ks values as well as, if available, the associated probability
Line 222: "These genes may have been intentional or inadvertent targets during domestication." Based on the presented data this is pure speculation and authors shouldn't build a case on these very limited data. My suggestion is to remove this sentence.
Lines 230-231: "The A and B subgenomes of teff have a near identical number of syntenic orthologs to Oropetium (19,277 vs. 19,292 respectively)". Are they also mostly the same genes?
Lines 232-233: "Orthologs to 1,308 Oropetium genes are found as single copy loci in teff, including 647 and 678 from the A and B subgenomes respectively". They Do not sum up to 1308 but 1325
Line 278: "homoeologous expression bias" Use HEB instead to be consistent.
Line 314: How much distant is "distant related"? Please put here some estimate of the divergence time between the two species.
Line 319: "Ph1-equivalent locus (loci)". Briefly explain.
Lines 360-369: It would be useful to add a supplemental figure clarifying the experimental design
Line 399: why a unimodal distribution does indicate high differentiation between the two subgenomes? I would have expected the opposite…
Line 416. Typo: "previuously" → "previously"
Lines 449-450: This has already been said in results, so it is redundant.
Line 473: specify the reference for the ratio you used.
Line 482. Why did authors use such an old version? This is just out of curiosity. With newest version result shouldn't change, though.
Lines 517-519: any estimate of the amount of genes excluded because of translocation?

Reviewer #2 (Remarks to the Author):

VanBuren and colleagues have produced a high-quality assembly and annotation of the polyploid and orphan crop, Teff. The data produced and basic analyses related to genome construction are of the

highest quality. However, I have a number of questions and concerns about the analysis and interpretation; these are detailed below.

Major comments:

1 … The title and take-home message of the manuscript regard excessive subgenome stability in Teff. The term exceptional implies that genomes of other species are far less stable. Despite detailed results of gene expression, large-scale collinearity, sequence similarity and TEs within Teff, there are few comparisons to the distribution of these parameters in other polyploids — so the reader is left to figure out on their own how exceptional Teff really is. For example, I'm not sure of what to make of the term 'high degrees of' synteny (line 313), since there are no baselines provided to compare to. Where examples are provided (e.g. Edger et al. 2017, line 329) the comparisons are limited to one or a few papers and not of comparably ancient polyploids.

2 … Lines 125 – 138 and 223 - 235: Using annotated sequences as a proxy for presence absence might be error prone – annotations are thresholded based on a variety of scores, so the presence of a gene but not its homeolog might be just as likely to be due to real sequence divergence as thresholding based on homology or expression support. Are you invoking pseudogenization as the primary mechanism here? If so, perhaps adding an analysis of the presence / absence of modeled pseudogenes or low-support gene models would help.

3 … Line 211 and elsewhere – what is meant by ortholog? Can't find mention in the methods about orthology inference. Just sequence similarity via last. Also, what is meant by syntenic? Just that the genes are in an mcscan block? If so, are all points in Fig. 4C considered syntenic? If so, what do you make of the apparent duplications (e.g. 1A/B(teff):6(oropetium)).

4 … I'd like to see more detail about how differential expression analyses were accomplished. Some things that should be addressed: 1) what statistical test, level of replication and software was used to determine HEB? — I think this is a test of allele-specific expression in 1:1 homeologs, but I am not sure how it was actually conducted. 2) How were genes with non-unique mapping handled? That is, were reads that mapped to both homeologs discarded (which might increase your ability to find HEB, and thus type1 error) or were they both counted? If the annotation was split into subgenomes before analysis, it might be worth exploring more nuanced allele-specific expression software like EMASE. 3) Its not clear how a wilcoxon test would be applied here – there is pseudo-replication at the level of homeolog among 207k non-unique comparisons (each gene is tested 10x). 4) How were multiple tests corrected for? It appears that 207k tests were made, but I imagine that FDR corrections were only employed within tissue type/treatment. Please detail how experiment-wise FDR was controlled.

--
Minor:

I don't understand the hypothesis explored in lines 257-259. Wouldn't homeologous exchange simply lead to multi-mapping reads (or identical expression if subgenomes mapped independently) and therefore not appear in HEB?

Historical patterns of selection are inferred both as sequence variation during domestication and in reference to HEB. These are accomplished without a lot of possible corrections for demography etc. I'd say these sections (lines 217:222, 286-291) could either be significantly bolstered by more nuanced analyses or presented as hypotheses. Plus, lines 217-222 don't really fit with the results having to do with subgenome differentiation that surround it.

I would disagree Fig 4C shows that teff and oropetium have near complete collinearity (Line 200). To me, this shows lots of inversions, translocations, etc. Furthermore, in the next sentence, it is stated that 13-15% of all genes do not display expected patterns of collinearity.

Since the article is about subgenome stability, I'd like to hear more about why copy number in arrays vary so extensively (Line 171) … more than other polyploid grasses with similar ages?

I was interested in the different sizes of the subgenomes, a fact is mentioned several times throughout; however, a quantification of the factors that lead to larger A subgenome is not accomplished, just general statements like that of line 189.

This is personal preference, but I found the genome assembly and annotation sections to be mostly methods with some summary statistics. To me, this is best summarized in a few sentences in the main text and expanded in the supplement (and methods), since these results do not address the central theme of the manuscript.

Reviewer #3 (Remarks to the Author):

Robert and co-authors sequenced the genome of allotetraploid teff, a type of cereal crop in Africa, through the combination of SMRT long reads and HiC data. They further separated the two subgenomes in the teff genome, and observed the homeoelog expression dominance between the two subgenomes. I have some suggestions for the authors to improve the manuscript before further consideration.

1. In the introduction section, it is introduced that the teff has "desirable nutritional profiles, abiotic and biotic stress resilience, and untapped genetic potential", and is "among the most resilient cereals, tolerating marginal and semi-arid soils". However, no such evidences have been provided in the analysis of the genome. The current manuscript lacks of biology research contents. Please focused on two or more biology features of teff, such as "nutritional profile" or "tolerating marginal and semi-arid soils", providing the genomic evidences and the underling biology stories.
2. The manuscript only focused on the genome sequencing, subgenome partition and homoeologs expression differentiation, while the evolution of the species to other cereal or close related species has not been provided. I suggest the authors to add these contents, such as phylogenetic tree, ka/ks among species, etc.
3. Authors discussed that the diploid ancestor of the teff might be extinct. However, no such analysis has been performed, authors should provide the evidence of the closest genomes to the two diploid ancestors, which may provide clues to trace the diploid ancestor of teff.
4. The authors discussed it that "wild progenitor of teff is likely Eragrostis pilosa", please provide some genomic evidences. Furthermore, the teff cultivar "Tsedey" was annotated to have 50006 genes, while the assembled teff has predicted 68,255 gene models. Why they are so different in gene numbers, what have been expanded, and how to evaluate the accuracy of the gene prediction process?
5. For the subgenome specific LTRs, they are very small in numbers, author identified 8-23 LTR copies for the six subgenome specific LTRs families. Please provide the statistic tests for such number of insertions occurred by random events.
6. The title name "Subgenome origins" should be changed to something like "subgenome partition…", since the origin of the two subgenomes has not been analyzed.
7. In lines 110, please explain why there are 32 linkage groups obtained from the population, which is much more than the number of chromosomes? How can you use this information and will not introduce extra errors?
8. Have the authors estimated the heterozygosity of the sequenced material, and how is the heterozygosity? Please provide this parameters.
9. In the abstract, "The exceptional subgenome stability" should not be the reason of "diversification of these grasses".

We thank the three reviewers for their constructive comments and their time in reading and critiquing our manuscript. We have carefully and thoroughly revised our paper based on these comments and we feel our revised manuscript is greatly improved. Below are our point by point responses to each comment.

Reviewers' comments:

Reviewer #1 (Remarks to the Author):

In this paper VanBuren et al present and describe a new genome assembly for the polyploid species Eragrostis teff. This chromosome scale genome assembly is based on PacBio reads along with HiC data and represents a great improvement in comparison to the previously available illumina based draft assembly (Cannarozzi et al, 2014). Indeed the statistics of completeness and contiguity for this assembly are impressive. Furthermore, using genome counterpart specific repetitive sequences the authors were able to associate each chromosome to the original donor genome (A or B). Analyzing the insertion time of TEs and their genome specificity the Authors were also able to estimate that the polyploidization event took place ~1.1 MYA. Analyzing the Ks between homeologus genes in E. tef the authors estimated that the two donor subgenomes diverged ~5.0 MYA. No major genome rearrangements were detected indicating an exceptional genome stability. Authors then provided an extensive survey of tef transcriptome showing divergent expression patterns among homeologous gene pairs. Definitely this is a great resource for those interested in tef and more generally in orphan crops. Also it is a nice piece of work providing interesting insights in polyploid genome structure and functioning.
Nevertheless there are few issues and a certain lack of detail that should be addressed by authors.

-First I would like to have the general statistics regarding repeats and TEs content and distribution moved to the main text (at present these data are provided in Supplemental Table 3). Indeed, as it clearly appears from the methods section, TEs annotation was carried out thoroughly but no general data are presented. Just a simple table clearly stating the amount of LTR-RT (possibly detailed for Ty1-copia and Ty3-gypsy), LINEs, SINEs etc… would be useful. I know that most part of this information is in main text and in supplemental data but I think that having it also in a single table in main manuscript would be beneficial for the readers.

We have moved Supplemental Table 3 to the main text so that the information about TEs is more accessible to the readers. We originally had this in the supplement because of space limitations.

-Then always related to TEs and in particular to those families seemingly specific for genome donors, all the period going from “We classified...” to “Supplemental table 5” (lines 177-192) is confusing and should be better described since it has a pivotal role in this manuscript. More in detail, it seems to me that authors dated the polyploidization event at 1.1 MYA (or before)

basically relying on two facts: 1) all the species specific families (six) should have amplified before polyploidization and 2) the estimated insertion time is > 1.1 MYA for all the elements belonging to these families. I do not know if I got the reasoning correctly here. If not please apologize and explain. Anyway if this is correct then there is another simple test that authors should carry out to further support their conclusion. Consider out of the “24 families with median insertion times between 1.1 and 2.4 MYA” those that “do not exhibit subgenomic specificity”. In this case, if you build a phylogenetic tree separately for PostPplyploidization (IT < 1.1 MYA) and PrePolyploidization (IT > 1.1 MYA) inserted elements then, for the first group elements should mix up whereas for the second subset they should separate in two distinct clades. Some families that could be considered for this exercise are 4, 6, 15, 16, 17, 25, 41, 43, 45, 52, 60 and 61.
This reasoning is correct, although there are no “species specific families”. There are families that were only active in one of the two species during the time the two ancestral species were separated (after divergence from a common ancestor). We provide some revised text on lines 192-196 that we believe makes this point more clearly. We did perform the phylogenetic tree analysis suggested by the reviewer. We used the reverse transcriptase (RT) sequence (because other LTR-retrotransposon genes were often truncated, and so we could get complete data for six of the suggested families that were not-truncated or completely missing: 6, 16, 43, 52, 60, and 61), and we used an Arabidopsis RT as the outgroup. For all six families, the trees matched the expected outcome in that the most recent transpositions (< 1.1 mya, clustering together in the trees) are found on both A and B chromosome sets. Those in the 1.1 to 5.0 mya window are not clustered with the younger elements (and are more diverse, of course, because of their age) and these 1.1 to 5 mya aged elements are found on only one subgenome, not both. We do not believe that the figures we generated for this analysis are appropriate for inclusion in the main body or supplementary portions of the manuscript, however, for two reasons. First, there were very few elements from these families that were active in the 1.1 to 5.0 mya window, so the average reader might find these results to be more confusing than illuminating, especially at the level of statistical significance. Second, the bootstrap values for many of these branches are less than 0.4 (presumably because of the short length of RT that was useable, ~220aa). Hence, we hope this analysis satisfies the reviewer but we do not believe it is needed for most readers, because of the statistical analysis we provide below.

Authors then stated that “The insertion times for these genome-specific LTR-RTs were all greater than 1.1 mya, indicating the two subgenomes were evolving independently during this period” but this doesn’t seem to be the case of Family 14 that shows evidence of recent activity (supplemental figure 4).

As noted by the reviewer, family 14 was active in both the pre-polyploid and post-polyploid time frame. This could be true for any family, and would not affect the conclusions drawn. We did not know the time between ancestral divergence and polyploid formation when we began this study, so we looked for LTR-RTs with medium or high copy numbers to see if there was any time period that they were subgenome specific in their insertions. We found six such families that showed subgenome-specific amplification during part of their evolutionary descent. All six were subgenome-specific during the 1.1 to 5.0 mya window, hence we conclude that this is the time

when the two genomes were separate in the two species that fused to form the polyploid. Many (presumably all) families also exhibited activity before the ancestral divergence, and some after ancestral divergence, but were not genome-specific in their insertions during those time periods. As noted above, we have changed the text somewhat on lines 197-200 to make this point more clear.

Also authors concluded that because “Five of the six subgenome-specific LTR-RT families were found only in the A subgenome” then “LTR-RTs accumulate more rapidly in the A subgenome or are purged more effectively in the B subgenome.” I am not completely sure about this. These are just subfamilies and authors are using their distribution pattern to infer the activity for the whole set of familyes: 65.

We fully agree with the reviewer that six data points (i.e., six families) is a bit thin to make this conclusion firmly, but we also feel it would be inappropriate to ignore the possibility. 5:1 is not a robust statistical number (p value >.2). So, we now tone this statement down in lines 208 and 209.

Other more specific points to fix or clarify are:

Line 84: is there a particular reason for focusing on “Dabbi” cv.? If there is, briefly explain it.

‘Dabbi’ is a high yielding landrace with historical significance, and has been used previously in the teff research community for both applied and basic research projects. We have included this in the paper (see line 98).

Lines 91-92: Authors should better specify the evolutionary relationship between E. tef and Oropetium thomaeum. How close is “closely related”?

Oropetium and Eragristis belong to different tribes within Chloridoideae (Cynodonteae vs Eragrostideae), so close is a relative term. We have removed ‘closely related’ and replaced it with ‘the Chloridoid grass Oropetium…”

Lines 99-102: from “ Illumina reads from...” to “...between homoeologous regions”. All this part should be moved to Materials and Methods

We have moved this section to the material and methods

Lines 115-117: It doesn’t seem that the incongruences in figure Figure 2, chr 8A are negligible. Quite likely they could be explained somehow... but “negligible” seems to me an understatement.

We agree with the reviewer that differences between the genetic map and pseudomolecules are not negligible, but are generally low. The small differences between the two are likely driven

primarily by a low rate of missing data and erroneous genotyping which can distort marker order. This is relatively common in GBS based maps. This genetic map was also constructed from an interspecific cross between *E. tef* and *E. pilosa*, and some of the small-scale differences may be driven by differences in physical sequence between the two species. The average Pearson correlation coefficient between marker and physical distance is quite high (0.932), and up to 0.98 for some linkage groups, suggesting errors in either the genetic map or pseudomolecules are minimal (but not negligible). We have updated the results and methods to reflect this.

Lines 142-144: These satellite sequences could well be centromeric satellite sequences. However it is important to be aware that the definition of centromeric sequence could not be based only on length and tandem arrangement. Here no prove is provided regarding the actual localization (FISH) and function (ad hoc biochemical essay) of these sequences although the analysis of co occurrence with centromeric specific Ty3-gypsy elements is a reasonable proxy.

For these reasons I think that these sequences are for sure satellite sequences but they are only putatively located in centromeres. Because of this I would suggest to change CentT in SatT.

We agree with the reviewer that without FISH or CENH3 ChIPseq, we cannot confirm that these satellite DNA sequences are the centromeric satellites. In the revised text, we refer to these as putative centromeric satellite sequences and have changed all instances of CentT to SatT as suggested by the reviewer.

Lines 148-149: “This element was previously identified in the draft teff genome”. Actually it was not identified in the draft teff sequence but in a set of random sheared MiSeq reads collected from a third Teff variety: Enantite

We thank the reviewer for catching this mistake, and have corrected it in the text.

Line 158: “The Teff subgenomes have 93.9% sequence homology”. It is not “93.9% homology” but “93.9% similarity”. Homology by definition means: “sharing a common ancestor” and so it can not be quantified as a percentage. It is there or it is not.

We have replaced ‘homology’ with ‘similarity’ in the text.

Lines 163-164: mention the mutation rate here (not just the reference)

We have added the substitution rate to the text.

Line 166: Table 1. In this table it would be nice to have listed also the TEs content for each chromosome.

We have added TE content for each chromosome in Table 1

Lines 177-178: “We classified LTRs into families” please explicit that these families are 65.

Corrected

Line 202: “Teff and Oropetium show a clear 1:2 synteny pattern” Shouldn’t it be the opposite, i.e. a 2:1 pattern?

Yes, corrected. We thank the reviewer for catching this typo.

Lines 217-221. Supplemental table 6. Why 0.38 was chose as cutoff? Also list in supplemental table 6 the actual Ka/Ks values as well as, if available, the associated probability

The ratio of > 0.38 represents the top 10% of genes with the highest Ka/Ks, so this cutoff was chosen based on percentile. Based on a suggestion from another reviewer, we have decided to remove this analysis from the text. These preliminary analyses are interesting, but require further downstream analyses. We feel the manuscript is already long and want to keep it focused on the findings on subgenome dynamics.

Line 222: “These genes may have been intentional or inadvertent targets during domestication.” Based on the presented data this is pure speculation and authors shouldn’t build a case on these very limited data. My suggestion is to remove this sentence.

We have removed this speculative sentence.

Lines 230-231: ”The A and B subgenomes of teff have a near identical number of syntenic orthologs to Oropetium (19,277 vs. 19,292 respectively)”. Are they also mostly the same genes?

Yes, 21,276 gene pairs are found in duplicate in teff and 1,325 are found in single copy in teff compared to Oropetium, so they are mostly the same genes. We have updated these numbers slightly (21,697 vs. 21,520) compared to (19,277 vs. 19,292). These original numbers were from an earlier version of the genome and were not updated in the original manuscript submission.

Lines 232-233: “Orthologs to 1,308 Oropetium genes are found as single copy loci in teff, including 647 and 678 from the A and B subgenomes respectively”. They Do not sum up to 1308 but 1325

We have updated this to 1,325, and this was an artifact of not updating the numbers from an earlier version of the genome (see above).

Line 278: “homoeologous expression bias” Use HEB instead to be consistent.

Corrected

Line 314: How much distant is “distant related”? Please put here some estimate of the divergence time between the two species.

Oropetium and teff belong to different tribes within Chloridoideae (Cynodonteae and Eragrostideae respectively) and diverged an estimated ~25 million years ago. The chromosome-scale collinearity is particularly striking given this divergence. We have added this to the text.

Line 319: “Ph1-equivalent locus (loci)”. Briefly explain.

The Ph1 locus in wheat enforces strict bivalent pairing and prevents homeologous exchange between the different wheat subgenomes. We hypothesize a similar locus (or loci) likely exist(s) in teff, and may be responsible for the total lack of multivalent pairing and homeologous exchange we observed. We have clarified this in text.

Lines 360-369: It would be useful to add a supplemental figure clarifying the experimental design

We have included a new supplementary figure (Figure S10) in the revision to show which tissues were used for the expression atlas. The tissues for developing seeds can be seen in Supplemental Figure 7.

Line 399: why a unimodal distribution does indicate high differentiation between the two subgenomes? I would have expected the opposite…

This is an interesting point. In an autopolyploid species, we would expect a bimodal peak of Ks distribution because distinct kmers between the two subgenomes (first peak) would have half the coverage of kmers that are the same between the two subgenomes (second peak). In an older allopolyploid (like tef), the subgenomes are so different that there would be few kmers shared between the two subgenomes, and we would observe only one peak of coverage corresponding to the expected genome size. Hopefully this is clear.

Line 416. Typo: “previuously” → “previously”

Corrected

Lines 449-450: This has already been said in results, so it is redundant.

We have removed this sentence

Line 473: specify the reference for the ratio you used.

We have added the reference

Line 482. Why did authors use such an old version? This is just out of curiosity. With newest version result shouldn't change, though.

This is simply an artifact of an old version that hasn't been updated. We agree with the reviewer that this will not affect the results.

Lines 517-519: any estimate of the amount of genes excluded because of translocation?

Given the high degree of collinearity, the fraction of translocations is likely low. Translocations of chromosome regions with > 5 genes would still be picked up using this synteny based approach. A gene family based approach would identify orthologs but it's difficult to say if these gene pairs are true 1:1 syntenic orthologs without detailed phylogenetic analyses.

Reviewer #2 (Remarks to the Author):

VanBuren and colleagues have produced a high-quality assembly and annotation of the polyploid and orphan crop, Teff. The data produced and basic analyses related to genome construction are of the highest quality. However, I have a number of questions and concerns about the analysis and interpretation; these are detailed below.

Major comments:

1 … The title and take-home message of the manuscript regard excessive subgenome stability in Teff. The term exceptional implies that genomes of other species are far less stable. Despite detailed results of gene expression, large-scale collinearity, sequence similarity and TEs within Teff, there are few comparisons to the distribution of these parameters in other polyploids — so the reader is left to figure out on their own how exceptional Teff really is. For example, I'm not sure of what to make of the term 'high degrees of' synteny (line 313), since there are no baselines provided to compare to. Where examples are provided (e.g. Edger et al. 2017, line 329) the comparisons are limited to one or a few papers and not of comparably ancient polyploids.

We agree with the reviewer that is an important point and the support for this claim of exceptional stability should be strengthened throughout the manuscript.
Other allopolyploid plants with similar origins and divergence times are much less stable in terms of chromosomal rearrangements, fractionation, and homeologous exchange. The cotton allotetraploid complex for instance, formed around the same time as teff (1.7–1.9 MYA), and the cotton A and D subgenomes diverged 6.2–7.1 MYA. The two subgenomes have several hundred megabases of translocated sequences and strucural rearrangements. This same

pattern of rearrangement is observed in the banana (*Musa balbisiana*) A and B subgenomes which diverged ~5.4 MYA and have several megabase pair sized translocations and inversions between them. The allohexaploid false flax (*Camalina stativa*) has evidence of ‘shattered’ chromosomes with numerous rearrangements and fractionation of the subgenomes compared to the diploid progenitors. Teff is unique compared to other ‘older’ allopolyploids as it has no-large scale structural rearrangements between the two subgenomes and a high proportion of genes are maintained in syntenic gene pairs. To date, this level of chromosome/subgenome stability has not been observed in similarly aged allopolyploids. We propose that the unusual stability of the teff subgenomes is due to suppressed homeologous exchange which prevents fractionation and rearrangements.

We have included these examples in the introduction and discussion to help support our claim of unusual subgenome stability.

2 … Lines 125 – 138 and 223 - 235: Using annotated sequences as a proxy for presence absence might be error prone – annotations are thresholded based on a variety of scores, so the presence of a gene but not its homeolog might be just as likely to be due to real sequence divergence as thresholding based on homology or expression support. Are you invoking pseudogenization as the primary mechanism here? If so, perhaps adding an analysis of the presence / absence of modeled pseudogenes or low-support gene models would help.

We agree with the reviewer, and this is an important point that has probably been overlooked in other polyploid genome projects. We searched for the missing homeologous genes using the set of low confidence gene models that were purged from the final annotation because of insufficient evidence or characteristics of pseudogenes. We also used BLAST to identify any genes that were fragmented, pseudogenized or were otherwise missed by MAKER. In total, we found evidence for 9,036 homeologous genes that were annotated as missing from one subgenome. Many of these genes had premature stop codons, were missing exons, or had no detectable expression, so most of these are presumably pseudogenes. It is possible that some ‘real’ genes failed to pass the threshold for inclusion in the final annotation or contain residual indel errors from the PacBio data. We have added this new analysis of homeologous gene pairs where one gene is still present as a likely pseudogene or e was missing from the MAKER annotation (see lines 174-178).
Taken together, this supports our finding of unusual subgenome stability in that most genes (~94%) are either found in high confidence syntenic gene pairs or have retained pseudogenes in one homeologous chromosome, and comparatively few genes (and their flanking regions) have been deleted. We have included this in the discussion.

3 … Line 211 and elsewhere – what is meant by ortholog? Can’t find mention in the methods about orthology inference. Just sequence similarity via last. Also, what is meant by syntenic? Just that the genes are in an mcscan block? If so, are all points in Fig. 4C considered syntenic? If so, what do you make of the apparent duplications (e.g. 1A/B(teff):6(oropetium)).

Orthologs are defined here as syntenic gene pairs in 2:1 synteny blocks (teff to Oropetium), with strict C-score cutoffs to eliminate retained whole genome duplicates from the shared rho and sigma WGD events in grasses. Given the strict cutoffs and clear 2:1 synteny, gene pairs are likely true orthologs and not other types of homologs.

Syteny is referring to gene pairs within syntenic blocks compared to Oropetium with the rho and sigma blocks (from the grass whole genome duplication events) filtered out using MCscan.

We have remade Figure 4c with stricter parameters to filter out residual hits from the shared grass whole genome duplication events. The apparent duplications between tef Chr 1A/B and Oropetium Chr 6 are from the rho WGD event and not syntenic orthologs. These residual gene pairs were filtered out in the subsequent analyses, but some were retained in this plot. We have updated the methods with the stricter C-score.

4 … I'd like to see more detail about how differential expression analyses were accomplished. Some things that should be addressed: 1) what statistical test, level of replication and software was used to determine HEB? — I think this is a test of allele-specific expression in 1:1 homeologs, but I am not sure how it was actually conducted. 2) How were genes with non-unique mapping handled? That is, were reads that mapped to both homeologs discarded (which might increase your ability to find HEB, and thus type1 error) or were they both counted? If the annotation was split into subgenomes before analysis, it might be worth exploring more nuanced allele-specific expression software like EMASE. 3) Its not clear how a wilcoxon test would be applied here – there is pseudo-replication at the level of homeolog among 207k non-unique comparisons (each gene is tested 10x). 4) How were multiple tests corrected for? It appears that 207k tests were made, but I imagine that FDR corrections were only employed within tissue type/treatment. Please detail how experiment-wise FDR was controlled.

HEB was identified between homeologous gene pairs using DESeq2 for all 10 samples in the teff expression atlas. Gene pairs were classified as having HEB if they passed the threshold of differential expression in DESeq2 using the model $y_{ij} \sim \mu + timepoint + e_{ij}$. Comparisons were made between the two homeologous gene pairs for each of the ten samples in the atlas. The built-in wald test in the DEseq2 package was used to test whether the log2fold change of a given gene was equal to 0 and genes with an fdr corrected, p-value < 0.05 were considered to be differentially expressed. We have added a paragraph this to the methods section (see Lines 528-534).

Homeologs have an average nucleotide identity of 93% with tight Ks distribution and little evidence of recent homeologous exchange, so read mis-mapping is not likely to be a major issue. Because of the relatively high divergence between homeologs, we used the full annotation from both subgenomes for read mapping and allele specific expression approaches were not necessary. The relatively high proportion of homeologous gene pairs classified as differentially expressed also supports accurate read mapping, because if reads were mapping to both homeologs we would expect (relatively) equal expression, and few genes classified as DE.

For the HEB comparisons, we were treating each pairwise comparison as a separate data point. The rationale behind this is some homeolog pairs have biased expression in both directions (toward the A and B homeolog) in different tissues so these aren't necessarily pseudoreplicates. Although we are testing each gene 10 times, we don't expect them to have the same pattern each time. We do agree with the reviewer that this is not the best way to test this, so we removed this test from the manuscript.

We instead tested each tissue separately. The HEB toward the B subgenome is significant for each of the 10 tissues (see line 260).

--

Minor:

I don't understand the hypothesis explored in lines 257-259. Wouldn't homeologous exchange simply lead to multi-mapping reads (or identical expression if subgenomes mapped independently) and therefore not appear in HEB?

Yes, recent homeologous exchanges would result in homeolog pairs with Ks close to 0, and multi-mapping reads, which would lead to more or less identical expression. Here, we are referring to older homeologous exchanges that occurred near the inception of the polyploidy event but have since diverged. These gene pairs would be different enough to have unique read mapping, but since they originated from the same subgenome, they would likely have similar expression levels, which would weaken patterns of subgenome dominance. This pattern has been observed in Brassica napus, Fragaria ananassa, and other polyploids.
We have revised this sentence for clarity.

Historical patterns of selection are inferred both as sequence variation during domestication and in reference to HEB. These are accomplished without a lot of possible corrections for demography etc. I'd say these sections (lines 217:222, 286-291) could either be significantly bolstered by more nuanced analyses or presented as hypotheses. Plus, lines 217-222 don't really fit with the results having to do with subgenome differentiation that surround it.

The Ka/Ks analyses revealed some interesting trends, but we agree with the reviewer that these findings are preliminary, and would be strengthened by additional, more detailed analyses. The manuscript is already quite long, and we feel including more analyses may take away from our central findings, and would be better suited for follow up work. Particularly regarding patterns of selection related to domestication, as large-scale resequencing of wild and domesticated teff is currently underway.
We have removed the analysis of genes under selection in teff given the preliminary and speculative nature of these findings. We kept in the analysis of Ka/Ks ratios for genes with HEB and added the hypothesis that homoeologous gene pairs with higher expression divergence are under more relaxed selective constraints.

I would disagree Fig 4C shows that teff and oropetium have near complete collinearity (Line 200). To me, this shows lots of inversions, translocations, etc. Furthermore, in the next sentence, it is stated that 13-15% of all genes do not display expected patterns of collinearity.

We have revised Figure 4C using stricter filtering to remove points corresponding to retained gene duplicates from the grass WGD events. We agree with the reviewer that there are clearly some inversions and translocations, but there are no chromosome-scale changes and fewer large-scale rearrangements than similarly divergent lineages. We have revised this sentence to state ‘a high degree of collinearity’ and go on to describe translocations or inversions between corresponding chromosomes of teff and oropetium. The near perfect macrosynteny of some chromosome pairs and relatively low number of inversions and translocations is unique in these lineages.

Since the article is about subgenome stability, I’d like to hear more about why copy number in arrays vary so extensively (Line 171) … more than other polyploid grasses with similar ages?

We hypothesize that tandem gene arrays are unassembled, underreported or unanalyzed in other polyploid grasses, and that differences in array sizes in teff are not unusual.  Significant differences in tandem gene content were observed between homeologous chromosomes of octoploid strawberry (see Edger et al. 2019), but these were not quantified in a pairwise manner. Tandem gene duplicates should be further analysed in other polyploid lineages to test whether the patterns observed in teff are unusual or a more universal feature of polyploids. Pan-genome analyses have uncovered vast differences in copy number variation between accessions of the same species, and the rapid birth/death of tandem gene duplicates likely drives the observed differences of array size in teff.

I was interested in the different sizes of the subgenomes, a fact is mentioned several times throughout; however, a quantification of the factors that lead to larger A subgenome is not accomplished, just general statements like that of line 189.

The differences in sizes are attributed to transposable elements. The A subgenome contains 35% more transposable elements than the B subgenome (87.5 vs 64.9 Mb) and the fraction of this repetitive DNA is higher in A than B. The density of genic regions is the same between the A and B subgenomes. We have added this to the results. The A subgenome is larger because it contains more repetitive DNA, but the reason why the A subgenome accumulates more (or fails to remove) repetitive DNA is unclear. We hypothesize that subgenome dominance and it’s associated relaxed selective constraints, differences in TE silencing/methylation, or rates of recombination related deletion may contribute to this pattern. This is highly speculative, but we do discuss this briefly in the manuscript.

This is personal preference, but I found the genome assembly and annotation sections to be mostly methods with some summary statistics. To me, this is best summarized in a few

sentences in the main text and expanded in the supplement (and methods), since these results do not address the central theme of the manuscript.

We agree with the reviewer that the genome assembly text is long and detailed, and we have moved portions of the text to the methods. We decided to leave most of the details in the main text because it outlines the unique steps we took to accurately assemble, phase, and quality check homeologous regions in teff. We feel this information is essential for the readers as it sets the stage for the interesting downstream analyses and could serve as a useful framework for others working on polyploid species.

Reviewer #3 (Remarks to the Author):

Robert and co-authors sequenced the genome of allotetraploid teff, a type of cereal crop in Africa, through the combination of SMRT long reads and HiC data. They further separated the two subgenomes in the teff genome, and observed the homeoelog expression dominance between the two subgenomes. I have some suggestions for the authors to improve the manuscript before further consideration.

1. In the introduction section, it is introduced that the teff has “desirable nutritional profiles, abiotic and biotic stress resilience, and untapped genetic potential”, and is “among the most resilient cereals, tolerating marginal and semi-arid soils”. However, no such evidences have been provided in the analysis of the genome. The current manuscript lacks of biology research contents. Please focused on two or more biology features of teff, such as “nutritional profile” or “tolerating marginal and semi-arid soils”, providing the genomic evidences and the underling biology stories.

We agree with the reviewer that the biological aspects of teff are certainly interesting and merit further functional genomics characterization. However, we feel this is largely beyond the scope of our manuscript. It is difficult to draw meaningful conclusions on agriculturally relevant traits based on genomic features alone. The integration of quantitative and population genetics, field trials, and physiology data would further our understanding of stress resilience in teff, but this is beyond the scope of our work. Our hope is that other researchers in the field, particularly those in Ethiopia, will use our foundational genomic resources in their research and breeding programs.

The primary biological focus of our manuscript are the unusual patterns of subgenome dynamics we observed in teff. We observed substantial homeolog expression bias across ten tissues, suggesting the two teff subgenomes have partitioned their ancestral functions. The most striking differences in homoeolog expression bias are observed during seed development and under abiotic stress. These patterns are likely linked to important agronomic traits and the natural resilience of teff. Despite ~5 million years of divergence, the teff subgenomes are highly collinear and most genes are retained. This stability contrasts other similarly aged allopolyploids

which show substantial gene loss, structural rearrangements, and homeologous exchange. The maintenance of two homeologous gene copies in the genome presents a significant obstacle for targeted breeding. We feel our manuscript has significant biological content beyond the genome, and our genomic resources will inform further downstream research in the important traits of teff.

2. The manuscript only focused on the genome sequencing, subgenome partition and homoeologs expression differentiation, while the evolution of the species to other cereal or close related species has not been provided. I suggest the authors to add these contents, such as phylogenetic tree, ka/ks among species, etc.

In the manuscript we have included detailed comparative genomics of teff and other Chloridoid grasses such as *Oropetium*. We have also included analysis of Ka/Ks ratios of genes in teff. The phylogeny of the major grass lineages is well supported by several studies including the first teff genome reported by Cannarozzi et al., and we don't think another tree would add much to our manuscript. We feel more detailed comparative genomics with other cereals is better suited for future work to avoid detracting from the central findings of our work.

3. Authors discussed that the diploid ancestor of the teff might be extinct. However, no such analysis has been performed, authors should provide the evidence of the closest genomes to the two diploid ancestors, which may provide clues to trace the diploid ancestor of teff.

The current consensus is that *Eragrostis pilosa* is the most likely direct wild progenitor of *E. tef,* and this is generally supported by karyotype, phylogenetic, and marker-based analyses. Our own work resequencing diverse teff accessions and numerous wild Eragrostis species supports this hypothesis as well, where phylogenetic, PCA, and STRUCTURE based analyses all show that *E. pilosa* is the most closely related wild species to *E. tef*. The teff polyploidy event occurred ~1.1 MYA (based on our dating), and is shared with tetraploid *E. pilosa*, suggesting polyploidy occurred well before the divergence of these two species and the domestication of teff. The two teff subgenomes are highly dissimilar as well, with an estimated divergence time of ~5.0 MYA. Together, this suggests that the diploid species are highly divergent and they merged a relatively long time ago (~1.1 MYA). Our own analyses and others in the field (see Ingram and Doyle, 2003) have failed to identify a diploid species with high similarity to one of the teff subgenomes. This could be due to poor taxon sampling and collections of putative ancestors from the teff center of origin, or it could be because the diploids have since gone extinct in the time since the initial polyploid event. We cannot rule out either of these possibilities and collecting more taxa to test this is beyond the scope of this manuscript. We agree with the reviewer that this is an important and interesting point and look forward to future efforts to identify the diploid progenitors and origins of the teff subgenomes.

4. The authors discussed it that “wild progenitor of teff is likely Eragrostis pilosa”, please provide some genomic evidences. Furthermore, the teff cultivar “Tsedey” was annotated to have 50006 genes, while the assembled teff has predicted 68,255 gene models. Why they are so different in

gene numbers, what have been expanded, and how to evaluate the accuracy of the gene prediction process?

Several previous studies have provided strong evidence that *E. pilosa* is likely the wild progenitor of teff based on phylogenetics (Ingram and Doyle, 2003), SSRs, and morphological characteristics. Furthermore, *E. pilosa* is the only Eragrostis species (to our knowledge) that is able to make fertile interspecific crosses with *E. tef*. Ingram and Doyle provide evidence that the allotetraploidy event in *E. tef* and *E. pilosa* is shared, but it is possible additional species were involved in the origins of teff but were not surveyed in this study. We did not collect the correct type of data to address this in our paper and analyses of subgenome differences and origins were done independently of assignment based on the parental genomes. In our resequencing work (described above), we have found strong evidence that *E. pilosa* is the likely progenitor of *E. tef*, but this project is in its early stages of analysis and is beyond the scope of this manuscript.

We included some analyses comparing the “Tsedey” and “Dabbi” genomes, but our analyses were fundamentally limited by the fragmented nature of the “Tsedey” reference and the incomplete annotation. Most homeologous genes in “Tsedey” were missing, and only 9,866 were found in pairs. Only ~30% of our gene models had homology to gene models in “Tsedey” and the remaining were either unannotated, or missing from the “Tsedey” genome. This is likely due to the highly fragmented nature of the Illumina based “Tsedey” reference. Because most of our gene models were missing from “Tsedey” we were unable to accurately quantify differences between the two accessions.

We assessed the annotation quality of the “Dabbi” reference using the Benchmarking Universal Single-Copy Ortholog (BUSCO) Embryophyta dataset. The annotation contains 98.1% of the 1,440 core Embryophyta genes and the majority (1,210) are found in duplicate in the A and B subgenomes. This supports that our annotation is high-quality. A high proportion of overlapping syntenic orthologs from other grass genomes also supports our annotation quality. We ran a BUSCO analysis on the “Tsedey” genome as well and found the annotation contained only 79.9% of the core Embryophyta genes and only 559 (38%) were found in duplicate in A and B copies. This supports that many essential genes are missing from “Tsedey”.

It would be interesting to compare gene presence/absence, copy number variation, and structural variation between the two genomes, but this will have to wait until the “Tsedey” genome assembly and annotation are improved.

5. For the subgenome specific LTRs, they are very small in numbers, author identified 8-23 LTR copies for the six subgenome specific LTRs families. Please provide the statistic tests for such number of insertions occurred by random events.

Very good and helpful point. The likelihood of an element exhibiting subgenome-specificity when it has 23 copies is 1 divided by 2 to the 22nd power, or less than .06%, but we looked at many LTR-retrotransposon families, so the likelihood of any showing subgenome specificity by chance is the sum of each individual chance, both for the elements that did show subgenome-

specificity and those that did not which we also investigated. However, no other LTR-RT families with transpositions of 8 or more were subgenome specific in any other time frame.

We only investigated LTR-retrotransposon families with intact element copy numbers of at least five so that we would not have low probability outcomes. With the lowest copy number of subgenome-specific LTR-RTs insertions that we found, in the 1.1 to 5.0 mya window, there were only 8 insertions, and these were all subgenome specific. This could have happened by chance for the 8 insertions at the frequency of 1 divided by 2 to the 7th power, or ~8%. All other subgenome specifics in these six families had a < 5% chance ($p < .05$) of occurring randomly. As mentioned above, there was no other timeframe in which any LTR-RT family with >7 insertions was subgenome-specific. In contrast, for all of the LTR-RT insertions of the six subgenome-specific families, the possibility of this happening by chance in that window is the multiple of each individual probability, or 1 chance in 2 to the 77th power, which is a very small number indeed. We now make these points more clearly on lines 192-202, providing statistical support for our argument.

6. The title name “Subgenome origins” should be changed to something like “subgenome partition…”, since the origin of the two subgenomes has not been analyzed.

We agree with the reviewer and have changed the title of this section to “Subgenome characteristics”

7. In lines 110, please explain why there are 32 linkage groups obtained from the population, which is much more than the number of chromosomes? How can you use this information and will not introduce extra errors?

Some of the chromosomes are split into multiple linkage groups because of insufficient marker data. We used the genetic maps as another line of evidence to support the accuracy of the HiC based pseduomolecules, and pseudomolecules were constructed independently of marker data. Because the genetic maps were only used as validation, they did not introduce errors into the assembly. The pseudomolecules and genetic map are highly collinear with an average Pearson correlation coefficient between marker and physical distance of $\rho=0.932$, which supports the accuracy of both datasets. Chromosomes that spanned multiple linkage groups were usually split at the centromere, and an example of this is shown in Figure 2. We have clarified this in the results.

8. Have the authors estimated the heterozygosity of the sequenced material, and how is the heterozygosity? Please provide this parameters.

We ran a kmer analysis to estimate the within genome heterozygosity of ‘Dabbi’. We observed a unimodal kmer peak around the expected diploid coverage, indicating the heterozygosity is quite low. The estimated heterozygosity was 0.15% based on this analysis.

9. In the abstract, “The exceptional subgenome stability” should not be the reason of “diversification of these grasses”.

We hypothesize that subgenome stability in teff and perhaps more broadly across Chloridoideae enables the ubiquitous polyploid formation within this subfamily. Polyploid lineages are associated with increased diversification rates and species richness (see Soltis et al. AJB, 2009 among others), and thus subgenome stability might enable polyploid which in turn could contribute to the diversification of Chloridoideae. Since this hypothesis has no empirical support, we have removed it from the abstract.

REVIEWERS' COMMENTS:

Reviewer #1 (Remarks to the Author):

I am satisfied with the explanations provided by authors and with the changes they made to the manuscript.
There are few minor modifications/corrections that I leave to the authors to carry out:

Typo: Camalina stativa → Camelina sativa

Table 2: "Family" better specify "Amount of families"

Table 2, For the SSR line, in columns "Subclass, Superfamily and Family" I would suggest to put "NA" ("Not applicable") since those three features refer to TEs

It seems to me that the numbering of Supplemental figures is offset by 1:

line 164: Supplemental figure 2b should be Supplemental Figure 1b

line 168 Supplemental figure 2a should be Supplemental Figure 1a

line 178 Supplemental figure 3 should be Supplemental Figure 2

line 201: supplemental figure 4 should be Supplemental figure 3

line 214: as line 201

line 236: Supplemental Figure 5 should be Supplemental Figure 4

line 269: Supplemental Figure 6 should be Supplemental Figure 5

and so on, I suspect. Please check.

Reviewer #2 (Remarks to the Author):

The authors have done a good job of addressing my concerns.

I appreciate the comparisons to similarly aged polyploids. It's not crucial, but I wanted to bring up that there are cases of similar complete synteny from much older 'diploidized' WGDs. For example, the Juglandaceae (e.g. https://www.nature.com/articles/s41438-019-0139-1) ... it appears that this polyploidy event is >60My old and synteny is nearly complete. I don't think this voids the idea that teff is 'exceptional', but could be brought up in the discussion if the authors felt it appropriate.

For completeness, I'd like to address some of the comments made in the response to reviews:

In the response to reviews, you present the presence/absence of homeologous exchange as a hypothesis, but in the main text it is presented as a tested result: '... we detected no evidence of recent homoeologous exchange' line 264 ... I don't see methods or results related to tests for suppressed homeologous exchange. Am I missing something? There are published tests for this using shared vs. unique kmers or snps.

In terms of the pseudogene discovery method outlined in the response to reviews; I see the results,

but I'd like a more detailed description of the methods in the main text. Like you say, this is an often overlooked test. Others will want to follow what was done here.

Reviewer #4 (Remarks to the Author):

Hopefully this will be clear in the web interface, but I am not reviewer #3 and was brought in specifically to review the points raised by Reviewer #3 and the author's responses to each point.

Reviewer 3, Point #1: My reading is that this may be a miscommunication to some extent between the reviewer and the authors and could be addressed by providing ciations to support the sentences on line 50-51 (characteristics of teff), 51-52 (grown primarily by subsistence farmers), 52-53 (number of cultivars developed), 53-54 (resilience of teff and tolerance for semi-arid soils) and/or omiting sentences from that set for which there are not good citations.

Point #2: I think the author's response here is fine. The phylogenetic placement of teff relative to other species with complete genome sequences is not at all ambiguous, and while studies of the placement of each subgenome relatve to other species in the genus Eragrostis might be interesting, in order to be novel they would require sequence from large numbers of genes in several of these species.

Point #3: The authors have provided a thoughtful response here about the two possible explanations: extinct diploid progenitors and incomplete sampling. Obviously it would never be possible to PROVE extinction but I would suggest that they also discuss the potential that this is simply an issue of incomplete sampling of related species. This could be as simply as another clause added on to the sentence on lines 66-68.

Point #4: Agreed. Comparing gene numbers between completely different structural annotation pipelines is always going to produce different numbers of gene models for technical, rather than biological, reasons.

Point #5: New text does a good job of addressing this concern.

Point #6: Change in title addresses this concern.

Point #7: Clarification in the text is good. I agree with the author's point, but as Reviewer #3's comment illustrates the reason for the mismatch between LGs and Pseudomolecules may not be apparent to many readers who have not previously worked in this area.

Point #8: Looks good.

Point #9: Authors have addressed it by removing this phrase.

REVIEWERS' COMMENTS:

Reviewer #1 (Remarks to the Author):

I am satisfied with the explanations provided by authors and with the changes they made to the manuscript.
There are few minor modifications/corrections that I leave to the authors to carry out:

Typo: Camalina stativa → Camelina sativa

Corrected

Table 2: “Family” better specify ”Amount of families”

Corrected

Table 2, For the SSR line, in columns “Subclass, Superfamily and Family” I would suggest to put “NA” (“Not applicable”) since those three features refer to TEs

Corrected

It seems to me that the numbering of Supplemental figures is offset by 1:

line 164: Supplemental figure 2b should be Supplemental Figure 1b

line 168 Supplemental figure 2a should be Supplemental Figure 1a

line 178 Supplemental figure 3 should be Supplemental Figure 2

line 201: supplemental figure 4 should be Supplemental figure 3

line 214: as line 201

line 236: Supplemental Figure 5 should be Supplemental Figure 4

line 269: Supplemental Figure 6 should be Supplemental Figure 5

and so on, I suspect. Please check.

Corrected

Reviewer #2 (Remarks to the Author):

The authors have done a good job of addressing my concerns.

I appreciate the comparisons to similarly aged polyploids. It's not crucial, but I wanted to bring up that there are cases of similar complete synteny from much older 'diploidized' WGDs. For example, the Juglandaceae (e.g. https://www.nature.com/articles/s41438-019-0139-1) ... it appears that this polyploidy event is >60My old and synteny is nearly complete. I don't think this voids the idea that teff is 'exceptional', but could be brought up in the discussion if the authors felt it appropriate.

We agree with the reviewer that there are likely other lineages with similar collinearity, but based on Figure 1B, there are dozens of large scale (> 5 Mb) rearrangements in *Juglans regia* including whole chromosome arms inverted between the ancestral subgenomes.

For completeness, I'd like to address some of the comments made in the response to reviews:

In the response to reviews, you present the presence/absence of homeologous exchange as a hypothesis, but in the main text it is presented as a tested result: '... we detected no evidence of recent homoeologous exchange' line 264 ... I don't see methods or results related to tests for suppressed homeologous exchange. Am I missing something? There are published tests for this using shared vs. unique kmers or snps.

We tested this using Ks differences between homeologous gene pairs in the teff A and B subgenomes. Regions with recent homeologous exchange would have significantly lower Ks and this would be maintained in windows spanning syntenic gene pairs. We did not observed large blocks of syntenic homeologous gene pairs with high similarity (low Ks) compared to the rest of the genome, indicating recent homeologous exchanges are not common in teff. We have added these details to the results and methods.

In terms of the pseudogene discovery method outlined in the response to reviews; I see the results, but I'd like a more detailed description of the methods in the main text. Like you say, this is an often overlooked test. Others will want to follow what was done here.

We have included the methods on how pseudogenes were identified.

Reviewer #4 (Remarks to the Author):

Hopefully this will be clear in the web interface, but I am not reviewer #3 and was brought in specifically to review the points raised by Reviewer #3 and the author's responses to each point.

Reviewer 3, Point #1: My reading is that this may be a miscommunication to some extent between the reviewer and the authors and could be addressed by providing ciations to support the sentences on line 50-51 (characteristics of teff), 51-52 (grown primarily by subsistence farmers), 52-53 (number of cultivars developed), 53-54 (resilience of teff and tolerance for semi-arid soils) and/or omiting sentences from that set for which there are not good citations.

We have added references to these sections and removed the claim that 130,000 locally adapted accessions have been developed because this is partially anecdotal.

Point #2: I think the author's response here is fine. The phylogenetic placement of teff relative to other species with complete genome sequences is not at all ambiguous, and while studies of the placement of each subgenome relatve to other species in the genus Eragrostis might be interesting, in order to be novel they would require sequence from large numbers of genes in several of these species.
We agree

Point #3: The authors have provided a thoughtful response here about the two possible explanations: extinct diploid progenitors and incomplete sampling. Obviously it would never be possible to PROVE extinction but I would suggest that they also discuss the potential that this is simply an issue of incomplete sampling of related species. This could be as simply as another clause added on to the sentence on lines 66-68.

We agree with the reviewer and have added this clause (unsampled) to the manuscript.

Point #4: Agreed. Comparing gene numbers between completely different structural annotation pipelines is always going to produce different numbers of gene models for technical, rather than biological, reasons.

Point #5: New text does a good job of addressing this concern.

Point #6: Change in title addresses this concern.

Point #7: Clarification in the text is good. I agree with the author's point, but as Reviewer #3's comment illustrates the reason for the mismatch between LGs and Pseudomolecules may not be apparent to many readers who have not previously worked in this area.
Clarified.

Point #8: Looks good.

Point #9: Authors have addressed it by removing this phrase.
